# Huanglian Jiedu Decoction improves the"central-peripheral"inflammatory microenvironment and enhances the cognitive function of APP/PS1 mice by inhibiting the activation of NLRP3 inflammasome mediated by gut microbiota

**DOI:** 10.1186/s13020-025-01180-4

**Published:** 2025-08-07

**Authors:** Yani Zhang, Jiahua Wang, Xuetao Li, Ruibo Guo, Liyan Wang, Yang Liu, Yang Yu, Liang Kong

**Affiliations:** 1 College of Pharmacy, Liaoning University of Traditional Chinese Medicine, Shengming 1 Road 77, Double D Port, Dalian, 116600 China; 2 Shenyang Key Laboratory of Chinese Medicine Targeted Delivery Key Laboratory, Shenyang, 110148 China

**Keywords:** Huanglian Jiedu Decoction, Alzheimer’s disease, Gut microbiota, NLRP3

## Abstract

**Background:**

Huanglian Jiedu Decoction (HLJDD) is a representative formula for clearing heat and removing toxins, and some basic studies indicated that it can improve the learning cognitive ability of Alzheimer’s disease (AD) mice, but the underlying molecular mechanism of its improvement in AD mice is still unclear, therefore, this paper delves into the mechanism of HLJDD to improve AD.

**Purpose:**

This study aims to investigate whether HLJDD can improve the “central-peripheral” inflammatory microenvironment in APP/PS1 mice, and to explore its relationship with gut microbiota and NLRP3 inflammatory vesicles activation.

**Materials and methods:**

In this paper, the fingerprint of HLJDD was established by high-performance liquid chromatography (HPLC) and the components of HLJDD were characterized by ultra-performance liquid chromatography-time-of-flight mass spectrometry (UPLC-O-TOF/MS). The potential signaling pathways of HLJDD against AD were preliminarily investigated through network pharmacology. Behavioral assessment, histopathological staining, immunofluorescence staining, immunohistochemical staining, and detection of central and peripheral inflammatory factors were used to explore the improvement of AD by HLJDD, in addition to which we examined the gut microbiota and expression of relevant inflammatory proteins.

**Results:**

In this study, 137 chemical constituents, including flavonoids, terpenoids, and alkaloids, were first identified in HLJDD by HPLC fingerprinting and UPLC-Q-TOF/MS. In addition, 49 components were found in the brain tissue of APP/PS1 mice and 48 components were found in the plasma of APP/PS1 mice. Network pharmacology concluded that the relevant pathways for HLJDD treatment of AD include inflammatory pathways. We found that HLJDD was effective in improving the learning memory ability of APP/PS1 mice by in vivo mouse behavioral performance. Histopathological results showed that HLJDD had the effect of reducing AD-like pathological damage, and also found that HLJDD could significantly reduce the proportion of M1 type microglia and A1 type astrocytes, and increase the proportion of M2 type microglia and A2 type astrocytes, and the treatment of HLJDD also suppressed the infiltration of CD4^+^ and CD8^+^ T-cells in the brain, and inhibited Aβ deposition and reduced the expression of inflammatory factors in the brain, and alleviated central neuroinflammation. In addition, it was also found that HLJDD was able to reduce the expression of inflammatory factors in the peripheral blood and inhibit the peripheral immune response, and the results of gut microbiota also showed changes in gut microbiota after HLJDD treatment and verified the expression of inflammatory vesicle-associated proteins in the intestines, with significant upregulation of the expression of NLRP3, caspase-1, and ASC proteins in the model group, and significant downregulation of ZO-1 and occludin proteins, and reversal of the above changes after HLJDD intervention.

**Conclusion:**

Therefore, it is hypothesized that HLJDD improves the “central-peripheral” inflammatory microenvironment in APP/PS1 mice by inhibiting the activation of NLRP3 inflammatory vesicles mediated by gut microbiota.

**Supplementary Information:**

The online version contains supplementary material available at 10.1186/s13020-025-01180-4.

## Introduction

Alzheimer’s disease (AD) is a common neurodegenerative disorder characterized by progressive memory and learning loss, mental behavioral abnormalities, and gradual loss of activities of daily living, which significantly affects the quality of life of patients and their caregivers [1]. Despite extensive research, the pathogenesis of AD has not been fully elucidated and there is no effective cure for the disease. The pathology of AD is closely related to a “central-peripheral” immune interaction network driven by neuroinflammation [2, 3]. Studies have shown that abnormal activation of central microglia or astrocytes and infiltration of peripheral immune cells form a vicious circle, constituting a continuously reinforcing neuro-immune loop, which serves as a catalyst for accelerating the pathological cascade of the Aβ/Tau core [4–6]. Moreover, under the continuous action of the above neurotoxic substances, progressive neuronal loss occurs in the hippocampus, which constitutes the pathological basis for the irreversible cognitive dysfunction in AD patients. In addition, neuroinflammation, oxidative stress and intestinal flora dysregulation also play important roles in the pathological process of AD [7]. Current therapeutic drugs for AD are mainly focused on relieving symptoms, but cannot effectively slow down the progression of the disease. Existing drugs can only act on a single target to improve the clinical symptoms of AD patients, and many of the adverse effects of the drugs gradually emerge with the prolongation of drug administration. However, traditional Chinese medicine (TCM) is often characterized by multi-pathway and multi-target action, which is more in line with the pathological mechanism of AD. Therefore, it is one of the most important ways to study the prevention and treatment of AD to give full play to the advantages of TCM and to actively explore effective formulas for the treatment of AD.

Huanglian Jiedu Decoction (HLJDD) is a representative formula for clearing heat and removing toxins, which is composed of four Chinese medicines: *Coptidis rhizoma*, *Scutellaria baicalensis*, *Phellodendri Huangbai* and *Gardenia jasminoides*, written by Ge Hong of the Eastern Jin Dynasty [8]. Current pharmacological studies of HLJDD show that it has antioxidant, anti-tumor, immunomodulatory, anti-inflammatory and intestinal flora regulating effects [9–12]. In the treatment of AD, studies have shown that neuroinflammation is another important pathological feature of AD in addition to amyloid plaque deposition and neuronal fiber tangles, which can not only exacerbate the pathological progression of AD and form a vicious circle with it, but also directly lead to synaptic dysfunction, neuronal apoptosis and vascular injury [13, 14]. Although basic research suggests that HLJDD may improve the learning and memory ability of AD mice and reduce pathological changes by reducing neuroinflammation in the brain, the potential molecular mechanism of the anti-inflammatory effect of HLJDD has not been further explored.

A growing body of research evidence emphasizes the critical role of the microbe-gut-inflammasome-brain axis in neurodegenerative diseases [15]. Although the impact of the gut microbiome in neurodegenerative diseases has been widely explored, the link between changes in gut microbiota and the gut-microbe-inflammasome-brain axis in AD patients lacks sufficient evidence. Studies have shown that gut microbiota can exacerbate inflammation through multiple pathways, such as direct triggering of inflammatory responses, generation of pro-inflammatory metabolites, and diminished immune regulation [16]. Amyloid and lipopolysaccharide (LPS) released by the gut flora can trigger intestinal and systemic inflammatory responses, which in turn induce neuroinflammation [17]. The NOD-like receptor protein 3 (NLRP3) inflammasome is important in the regulation of host physiology and neurologic disease The NLRP3 inflammasome plays an integral role in regulating peripheral and central inflammatory responses in host physiology and neurological disorders [18]. Upon sensing microbial or danger signaling molecules, the pyrin structural domain of NLRP3 interacts with the pyrin structural domain of apoptosis-associated speckled protein (ASC) to initiate the formation of the inflammatory complex consisting of NLRP3, ASC, and caspase-1 [19]. Activation of this complex promotes the secretion of the pro-inflammatory IL-1β and IL-18 and induces cellular focal death. A growing body of evidence reveals a complex interaction between the gut microbiota and NLRP3 inflammasome. In this study, we will delve into whether Huanglian Jiedu Decoction inhibits inflammatory vesicles by modulating the gut microbiota, thereby ameliorating cognitive dysfunction in mice.

## Materials and methods

### Materials and reagents

Huanglian, Huangqin, Huangbai and Zhizi were purchased from the Department of Pharmacy of Liaoning University of Traditional Chinese Medicine Hospital, ELISA inflammatory factor kits were purchased from Solarbio Science and Technology Co. Ltd. (Beijing, China), HE kit was purchased from Meilun Biotechnology Co. Ltd. (Dalian, China), ZO-1, iNOS and ACS antibodies were purchased from Affinity Biosciences Ltd. (Jiangsu, China), Occludin, NLRP3, Caspase-1, GFAP, Arg-1, S100A00 and ASC antibodies were purchased from BOSTER Biological Technology Co. Ltd. (Wuhan, China). C3 antibody was purchased from Proteintech Group, Inc (Wuhan, China). Iba-1 antibody was purchased from FUJIFILM (Tokyo, Japan). Horseradish peroxidase (HRP) biotin-labeled secondary antibody was purchased from Biosynthesis Biologicals (Beijing, China), and HE and Nissl staining kits were purchased from Meilun Biotechnology (Dalian, China).

### Preparation of Huanglian Jiedu Decoction

Huanglian, Huangqin, Huangbai and Zhizi according to the ratio of 3:2:2:3 crushed into small pieces after mixing with 10 times the amount of distilled water resuspension immersion 40 min plus 10 times the amount of water decoction 1.5 h, filtration to take the filtrate, dregs of the medicine again with 8 times the amount of distilled water decoction 1.5 h, filtration to take the filtrate, the two filtrate combined with decompression and concentrated to 0.2 g/mL, − 20℃ freeze-drying to powder, to obtain the HLJD water extract.

Fingerprints of the extract of HLJDD were established by HPLC, and the chemical constituents of HLJDD were identified in vivo and ex vivo by UPLC-Q-TOF/MS. Preparation of serum samples from APP/PS1 mice, six APP/PS1 mice were given 8 g/kg/d of HLJDD for 3 consecutive days, and the serum samples were collected before and at 1, 2, and 3 h after administration, and kept in a refrigerator at −80 °C. Brain tissue samples of APP/PS1 mice were prepared in the same way as above, and the supernatant of brain tissue homogenate was taken and stored at −80 °C in a refrigerator.

### Animals

APP/PS1 mice were used as the animal model of AD, and randomly divided into model group, low-dose HLJDD extract group (2 g/kg), medium-dose HLJDD extract (4 g/kg), high-dose HLJDD extract (8 g/kg), and donepezil hydrochloride (2 mg/kg) groups, with 12 mice in each group, and the treatment groups were gavaged with different doses of Huanglian detoxifying tonic extract or donepezil hydrochloride once a day for 8 weeks. In addition, 12 wild-type C57BL/6 mice of the same age were taken as control group. The control group and the model group were given equal volumes of saline. Brains and intestines were removed and stored at − 80 °C after mouse execution, and 4-week-old APP/PS1 mice were purchased from Liaoning Changsheng Biological Co. All the experiments were conducted in accordance with the guidelines of the Institutional Animal Protection and Use Committee of Liaoning University of Traditional Chinese Medicine.

### Network pharmacology analysis

The blood and brain entry components of HLJDD were analyzed using UHPLC-Q-Orbitrap HRMS. The SMILES structural information was obtained from the PubChem database. Subsequently, potential action targets were predicted using the SwissTargetPrediction database, where targets with probabilities greater than 0 were included. Duplicate data were corrected and removed using the UniProt database. To identify the common targets between HLJDD and AD, the GeneCards, OMIM, and TTD databases were systematically searched. AD-related targets were retrieved using the keyword"Alzheimer’s Disease,"and key overlapping targets were visualized using Venn diagrams after intersection with HLJDD targets. A “drug component—target—disease” network was constructed using Cytoscape 3.9.1 software to illustrate the potential relationships between the active components of HLJDD and AD treatment. The intersection targets of HLJDD for AD treatment were then imported into the STRING database to construct the Protein–Protein Interaction (PPI) network. After removing free nodes, network topology was analyzed with Cytoscape 3.9.1, and the topological parameters of the network nodes were evaluated using the centiScape plugin. Based on three parameters: betweenness centrality (BC), closeness centrality (CC), and degree, the medians of these indicators were calculated. Targets exceeding the corresponding median values were predicted as core targets. Finally, targets were ranked based on their degree values. The DAVID 6.8 database was utilized for Gene Ontology (GO) and Kyoto Encyclopedia of Genes and Genomes (KEGG) enrichment analyses on the potential targets, with a screening criterion set at P ≤ 0.05. The top 10 GO and 20 KEGG enrichment analysis results that met the criteria were visualized.

### Behavioral assessment

#### Morris water maze

The Morris water maze test and nesting test were used to assess the learning memory ability and behavioral performance of mice. In the Morris water maze test, mice were trained for 60 s per day for 5 consecutive days, and their avoidance latency to find the hidden platform in four quadrants was recorded. If the mice failed to locate the platform within the allotted time, they were guided to stay for 10 s to reinforce the memory. At the end of the training, the platform was removed and a 60-s spatial exploration test was performed to analyze the number of times the mice entered the target quadrant using an automated tracking system.

#### Nesting score

The daily behavioral performance of the mice was assessed by a nesting experiment, the mice were individually housed and provided with five 5 cm × 5 cm cotton pads, and nesting was recorded and quantitatively scored at different time points. The nesting score of the mice was evaluated according to the previous study [20].

### Histopathological staining

For the brain tissues of mice, HE staining and Nissl staining were used to observe the structures of cortical neurons, CA1 neurons and hippocampal neurons in APP/PS1 mice with different formulas, and immunohistochemical staining was used to detect the expression of Aβ protein, CD4^+^T and CD8^+^T cell infiltration. In addition, intestinal tissues of mice were collected. The pathological changes of intestinal tissues were detected by the HE staining and the expressions of ZO-1 and Occludin were detected by immunohistochemistry.

### Immunofluorescence staining

Brain tissues were deparaffinized for antigen repair, closed with 5% BSA for 1 h, and then incubated with primary antibodies Iba-1, iNOS, Arg-1, GFAP, C3 and S100A10 overnight at 4 °C, followed by incubation with secondary antibodies FITC/Cy3 for approximately 1 h, restaining with DAPI, and imaging.

### Inflammation related indicator tests

Peripheral blood samples from mice were collected through eyelid blood sampling. After centrifugation at 3000 rpm for 10 min, the serum was separated. Pre-cooled PBS was added to the brain tissue to prepare a homogenate, which was subsequently centrifuged at 3000 rpm for 10 min to collect the supernatant. The levels of IFN-γ, IL-4, IL-6, and IL-1β in both peripheral blood and brain tissue were measured using kits.

### Routine blood analysis

The experimental mice were anesthetized and fixed on the operating table, the hearts were stripped, the right atrium was punctured quickly with a 1 mL syringe, and the blood was slowly aspirated 0.8–0.9 mL. The blood was injected into EP tubes containing EDTA-K2 anticoagulant, and then shaken well, and the proportions of leukocytes, neutrophils, lymphocytes, monocytes, eosinophils, and basophils were analyzed by using the fully automated hemocyte analyzer.

### 16S rRNA sequencing of the fecal microbiota

Fecal microbial community composition was analyzed using 16S rRNA sequencing. Total DNA from fecal samples was first extracted and PCR amplified, followed by high-throughput sequencing using the Illumina Novoseq6000 PE250 platform. The obtained sequencing data were processed by QIIME software, and operational taxonomic units (OTU) cluster analysis and species taxonomy analysis were performed using the UCLUST sequence alignment tool with 97% similarity as the threshold.

### Molecular docking

The three-dimensional structure of the core active ingredient of HLJDD was obtained from the PubChem database. This structure was subsequently imported into ChemBio3D Ultra 14.0 for energy minimization, after which the small molecule was saved in mol2 format. The optimized small molecules were then imported into AutoDockTools v1.5.6 for hydrogenation, charge calculation, charge allocation, and specification of rotatable bonds, before being saved in pdbqt format. Additionally, the two-dimensional structure of the NLRP3 and Caspase-1 protein was downloaded from the PDB database. The protein structure was imported into PyMOL 2.3.0 to remove crystalline water and original ligands. Following this, the protein structure was imported into AutoDockTools (v1.5.6) for hydrogenation, charge calculation, charge allocation, atomic type specification, and was saved in pdbqt format. The binding sites of the protein were predicted using POCASA 1.1, and docking was performed using AutoDock Vina 1.1.2.

### Western blot

Western blotting was used to detect NLRP3 inflammasome-associated protein expression in intestinal tissues. Intestinal tissue samples stored at −80℃ were taken, and RIPA lysis solution was added to extract the total protein, and the supernatant was collected after centrifugation at 4 ℃ for 20 min, and the protein concentration was determined by BCA method and boiled to denaturation. Appropriate amount of denatured protein sample was taken, separated by electrophoresis and transferred to membrane, and 5% skimmed milk was closed for 2 h at room temperature. Add NLRP3, Caspase-1, ASC, ZO-1 and occludin primary antibody (1:1000, v:v) respectively, and incubate at 4℃ overnight, then add the corresponding secondary antibody (1:1000, v:v) and incubate at room temperature for 1 h. Finally, ECL chemiluminescent solution was added dropwise to develop the color, and the images were captured by chemiluminescent imaging system and quantitatively analyzed by gray value using Image J software.

### statistical analysis

Statistical analysis was performed using GraphPad Prism 9.0 software, and data were expressed as mean ± SD. Comparisons between groups were analyzed by one-way ANOVA. The escape latency was analyzed by repeated measures two-way ANOVA with Dunnett post-hoc test. Results are presented as mean ± standard error of mean (SEM), with *P* < 0.05 indicating a statistically significant difference.

## Results

### Studies on the pharmacological substance basis of HLJDD

**Fig. 1:**
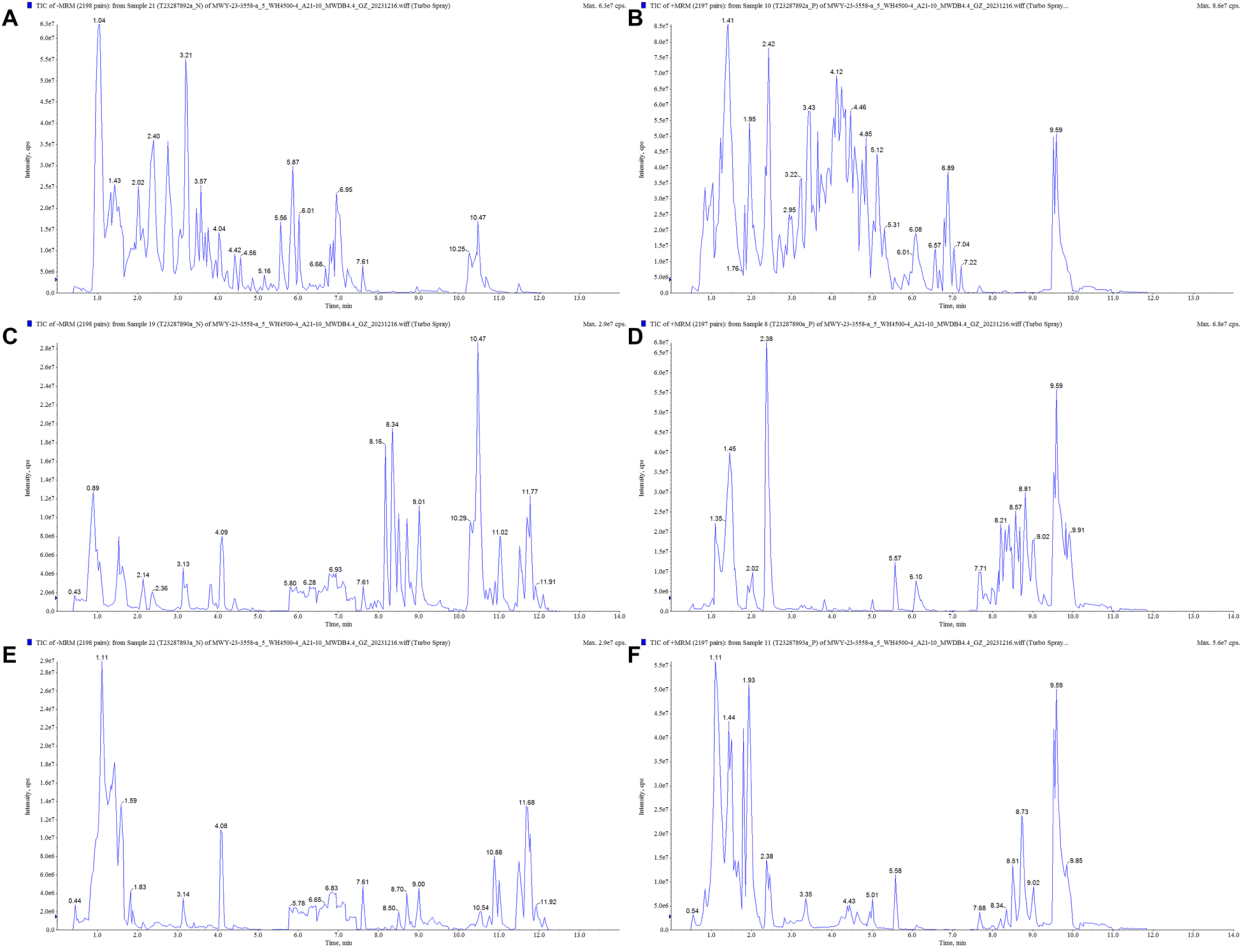
Chemical composition of HLJDD was determined.** A** TIC diagram of negative ion mode of HLJDD. **B** TIC diagram of positive ion mode of HLJDD. **C** TIC diagram of drug-containing serum negative ion pattern. **D** TIC diagram of drug-containing serum positive ion pattern. **E** TIC map of negative ion pattern in brain tissue. **F** TIC map of positive ion patterns in brain tissue

In order to deeply explore the material basis of the anti-AD efficacy of HLJDD, we performed UPLC-MS/MS analysis with gradient elution using 0.1% formic acid aqueous solution-0.1% formic acid acetonitrile solution as the mobile phases, and full scans of the extract of HLJDD, brain tissue of APP/PS1 mice, and plasma of APP/PS1 mice were carried out in both the positive and negative ionization modes, and the sample base-peak ion the total ion current (TIC) chromatograms are shown in Fig. 1A–F. A total of 137 chemical constituents were identified from HLJDD (Table. S1), including 51 flavonoids, 22 terpenoids, 20 alkaloids, 24 organic/phenolic acids, 6 lignans/coumarins, and 14 other compounds. Of these, 49 components were found in the brain tissue of APP/PS1 mice (Tables S2), 48 components were found in the plasma of APP/PS1 mice (Table S3). And 42 of these compound components were simultaneously found in the blood and brain tissues (Table S4).

### Network pharmacology study of HLJDD on APP/PS1 mice

**Fig. 2:**
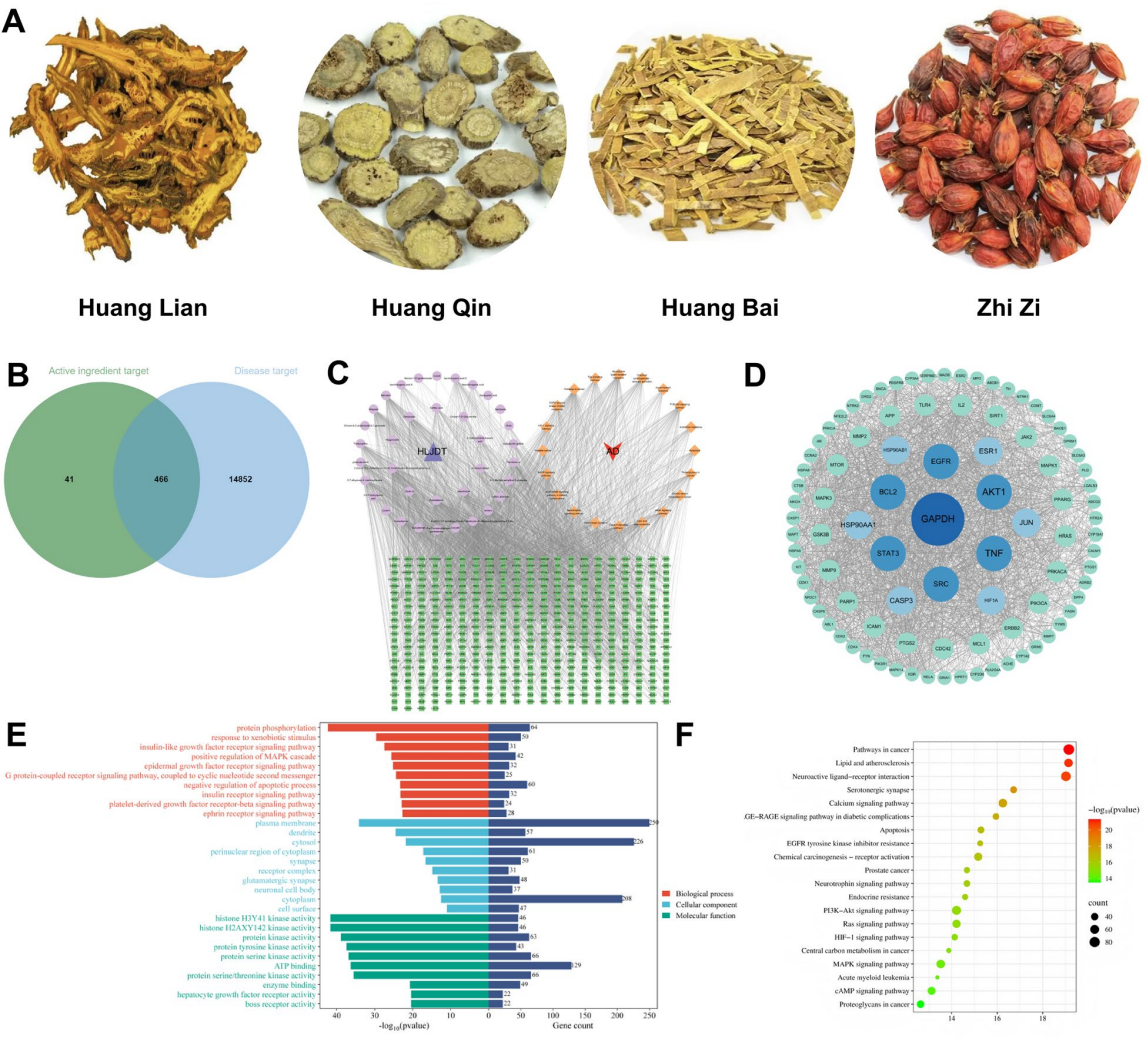
Study on network pharmacology of HLJDD against APP/PS1 mice. **A** Images of the various medicinal materials of HLJDD. **B** Venn plot of the intersection target of HLJDD and AD, where the green circles represent the active ingredient targets, and the blue circles represent the targets related to AD.** C** Image of the blood-entry and brain-entry component-target—disease pathway of HLJDD. The circles represent the blood-entry components, the rhombus represents the related pathways, the square represents the intersection targets, the triangle represents HLJDD, and the inverted triangle represents AD.** D** PPI network diagram of the core target. **E** Analysis diagram of the potential target GO pathway of HLJDD in the treatment of AD. **F** Bubble plot of KEGG pathway analysis for potential targets of HLJDD in the treatment of AD

**Fig. 3:**
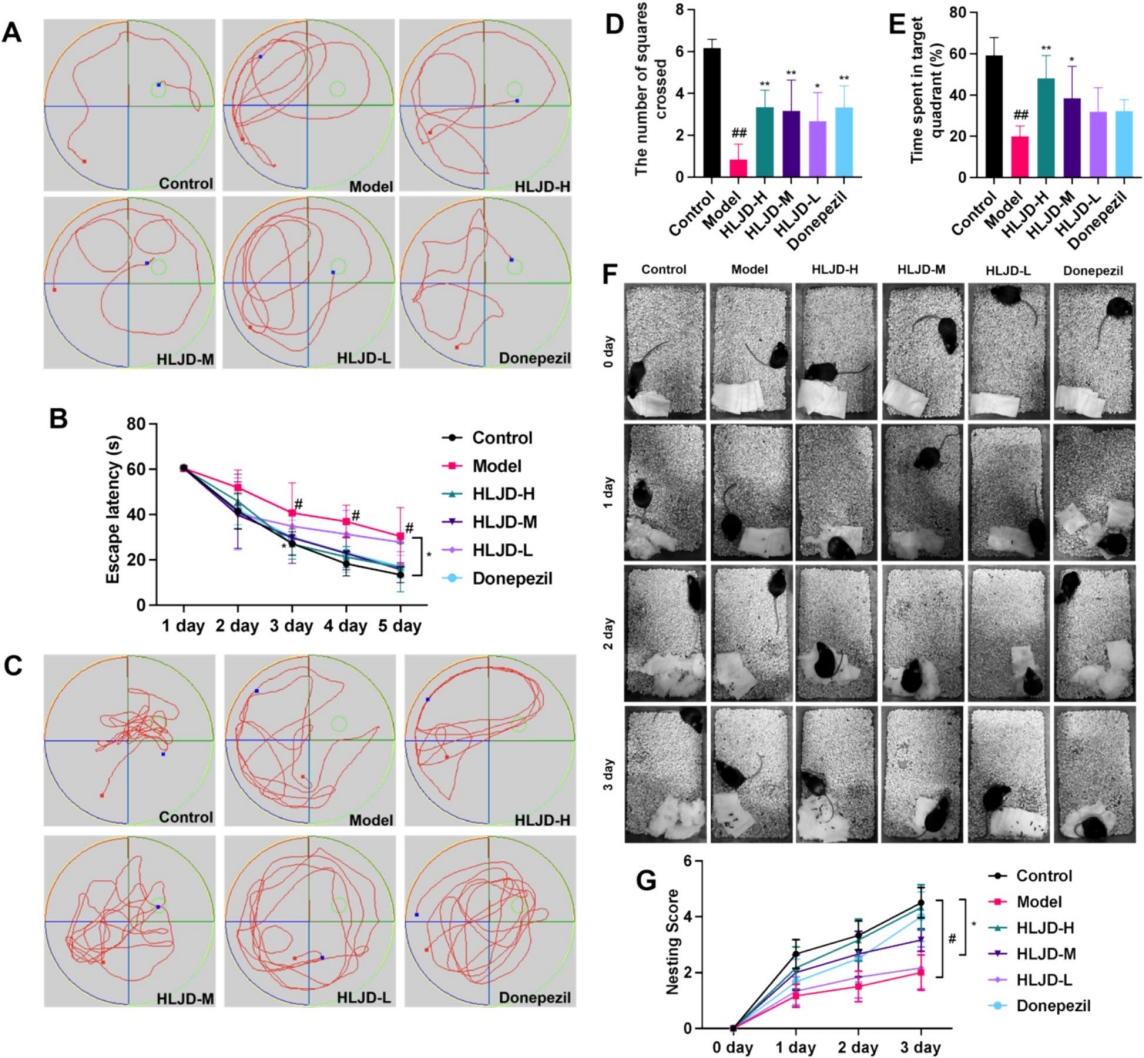
Effects of HLJDD on cognitive function in APP/PS1 mice. **A** Morris water maze was used to detect behavioral changes in mice. **B** Escape latency analysis of mice in each group. **C** The swimming trajectory of the mice on the 6th day within 60 s.** D** Data analysis of the time spent in the target quadrant on the sixth day. **E** The number of platform crossings in mice on the sixth day. **F** Representative images of the nesting experiment of mice from 0 to 3 days. **G** The quantitative analysis results of the nesting score. Data are presented as mean ± SD and data are presented as mean ± SD (n = 6). ^##^*P* < 0.01 (vs control group), ^*^P < 0.05 and ^**^P < 0.01 (vs model group)

To preliminarily explore the mechanism of action of HLJDD in the treatment of AD, we employed network pharmacology to predict potential pathways of action (Fig. 2A). A total of 36 components that can enter the bloodstream and brain were selected, excluding six components that lacked potential targets. We identified 507 targets associated with these components and 15,318 targets related to AD, resulting in the identification of 466 overlapping targets (Fig. 2B). The component-target-pathway map was constructed using Cytoscape 3.9.1 software. In Fig. 2C, circles represent the components, rhombuses denote the related pathways, squares indicate the intersection targets, triangles represent HLJDD, and inverted triangles signify AD. We predict that the core components with significant therapeutic potential include: (12bs)−4,10,11-trimethoxy-7,8,12B,13-tetrahydro-5 h-6-azatetraphen-3-ol, Baicalein, Epiberberine, 3’,7-dihydroxy-4’-methoxyflavone, Thaliporphine, Wogonin, Kumatakenin, Jatrorrhizine, 6-ethyl-1,10-dimethoxy-5,6,6A,7-tetrahydro-4H-dibenzo[de,g]quinoline-2,9-diol, phellodendrine, and Hamiltone A. The 466 overlapping targets of HLJDD and AD were input into the STRING database to generate a protein–protein interaction (PPI) graph comprising 92 nodes and 1,947 edges (Fig. 3D). The key intervention targets primarily include GAPDH, AKT1, TNF, SRC, EGFR, BCL2, STAT3, HSP90AA1, CASP3, and JUN, et al. (Table S5).

In the GO functional enrichment analysis, a total of 1,031 biological processes (BPs) may be influenced by HLJDD. These processes primarily include protein phosphorylation, response to xenobiotic stimuli, insulin-like growth factor receptor signaling pathways, positive regulation of the MAPK cascade, epidermal growth factor receptor signaling pathways, and G protein-coupled receptor signaling pathways coupled to cyclic nucleotide second messengers. Additionally, there are 137 cellular components (CCs) identified, which mainly encompass the plasma membrane, dendrites, cytosol, perinuclear region of the cytoplasm, synapses, and receptor complexes. The Molecular Functions (MF) category consists of 354 items, including histone H3Y41 kinase activity, histone H2AXY142 kinase activity, protein kinase activity, protein tyrosine kinase activity, protein serine kinase activity, and ATP binding. The results of the KEGG pathway enrichment analysis indicated that there are a total of 181 pathways relevant to treating AD with HLJDD. The enrichment analysis results of the top 20 KEGG pathways were input into microbiome bioinformatics data analysis platform, generating a bubble chart for KEGG pathway enrichment analysis. From Fig. 2F, it is evident that the primary signaling pathways involved in the treatment of AD by HLJDD include pathways in cancer, lipid and atherosclerosis, neuroactive ligand-receptor interactions, serotonergic synapses, the PI3K-Akt signaling pathway, the Ras signaling pathway, and the MAPK signaling pathway.

### Effects of HLJDD on cognitive function in APP/PS1 mice

In the present study, the effects of HLJDD on learning memory ability of AD model mice were evaluated by Morris water maze experiment. The results showed that compared with the blank group, mice in the model group and the HLJD-L exhibited significant spatial cognitive deficits manifested by disturbed motor trajectories (Fig. 3A), prolonged avoidance latency (Fig. 3B), as well as shortened residence time in the target quadrant and reduced number of traversing platforms (Fig. 3C–E). On the other hand, mice in the positive drug group, HLJD-M and HLJD-H showed significant improvement in cognitive function, and their behavioral performance was similar to that of the control group, which was characterized by a more concise motor trajectory, significantly shorter evasion latency, and the ability to maintain the memory of the original platform position after the platform was removed. These results suggest that the spatial learning and memory ability of APP/PS1 mice can be effectively improved by HLJD-M and HLJD-H.

We further used the nesting experiment to assess the cognitive function of AD mice. The experimental results showed that during the 24-h observation period, the mice in each group showed only a small amount of action. At 48 h, mice in the control group, the positive drug group, HLJD-H and HLJD-M were able to complete the initial construction of their nests and maintain their structural integrity, and at 72 h, mice in the control group, the positive drug group, the HLJD-M group and the HLJD-H group successfully constructed well-constructed nests (Fig. 3F), and the results of the nest-building scores were in line with the observed phenomena (Fig. 3G). The combination of the results of the water maze and nest building experiments confirmed that HLJDD treatments significantly improved the cognitive dysfunction of the AD model mice, allowing them to regain near-normal levels of cognitive ability after a short period of training. This finding further supports the ameliorative effect of HLJDD on cognitive impairment in AD.

### Effects of HLJDD on hippocampal neural structure and Aβ deposition in APP/PS1 mice

**Fig. 4:**
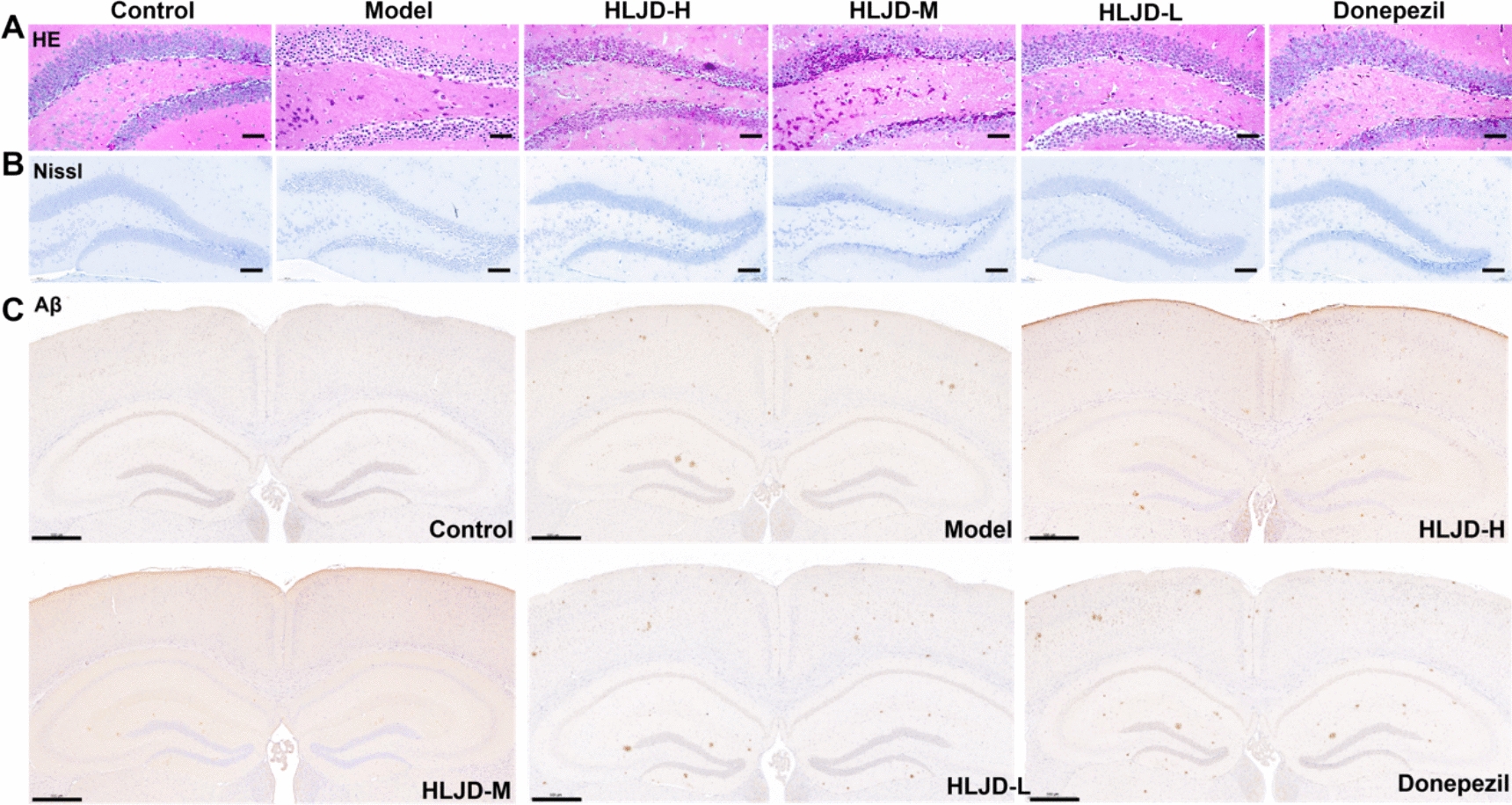
Neuroprotective effect of HLJDD on APP/PS1 mice. **A** Representative pictures of HE staining of brain tissue in mice, scale bar = 100 μm (n = 6). **B** Representative pictures of Nissl staining of brain tissue in mice, scale bar = 100 μm (n = 6). **C** Representative pictures of Aβ deposition of brain tissue in mice, scale bar = 500 μm (n = 6)

Histopathological analysis revealed the mechanism of action of HLJDD on neuroprotection in AD model mice. HE staining results showed that the cortex, hippocampus and CA1 area of the model group mice presented typical features of neuronal damage, which was manifested as cellular crumpling and sparse arrangement. After HLJDD intervention, neuronal morphology and distribution were significantly improved (Fig. 4A). The results of Nissl staining showed that HLJDD had no significant promoting effect on hippocampal neurons and hippocampal neurogenesis (Fig. 4B). Immunohistochemical analysis showed that HLJDD significantly reduced Aβ plaque deposition in the whole brain and hippocampus of APP/PS1 mice, with the most significant improvement in the HLJD-M and HLJD-H groups (Fig. 4C). These results suggest that HLJDD may exert neuroprotective effects through multiple pathways such as attenuating neuronal damage and inhibiting Aβ deposition, thereby improving AD-related pathological features.

### Effects of HLJDD on central neuroinflammation in APP/PS1 mice

**Fig. 5:**
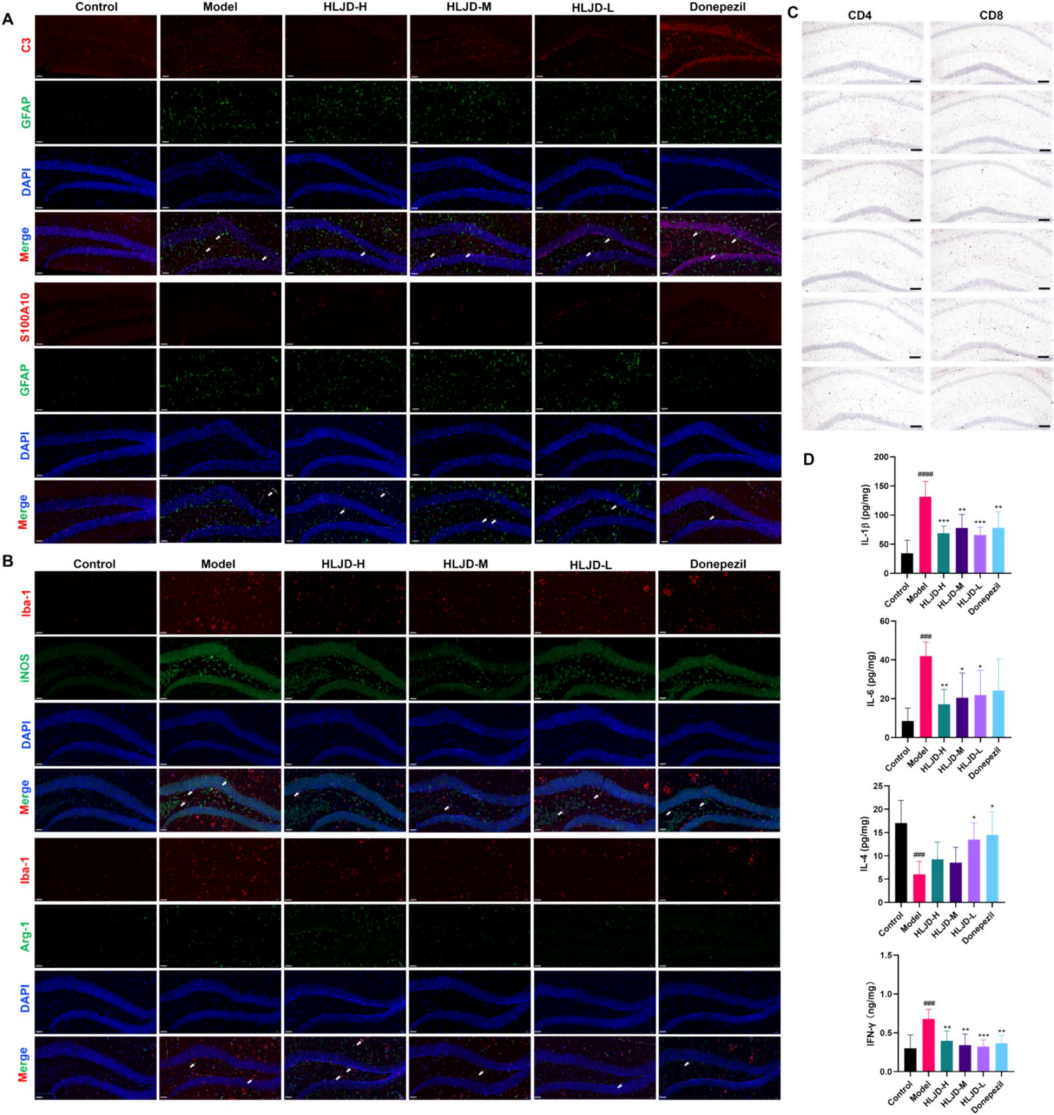
Effects of HLJDD on central neuroinflammation in APP/PS1 mice. **A** Expression of GFAP/C3 and GFAP/S100A10 in hippocampus of mice in each group (The white arrow indicates double positive staining). **B** Expression of Iba-1/Arg-1 and Iba-1/iNOS in hippocampus of mice in each group (The white arrow indicates double positive staining). **C** The infiltration of CD4^+^T and CD8^+^T cells in hippocampus of mice in each group. **D** Levels of IL-1β, IL-6, IL-4, and IFN-γ in brain tissue. Data are presented as mean ± SD (n = 6). scale bar = 100 μm. ^##^*P* < 0.01 and ^###^*P* < 0.001 (vs control group), ^*^P < 0.05, ^**^P < 0.01 and ^***^P < 0.001 (vs model group)

Astrocytes and microglia are crucial immune cells in the central nervous system, playing a pivotal role in the regulation of neuroinflammation. To investigate the effects of HLJDD on these two cell types, we employed immunofluorescence staining techniques to assess its regulatory effects on the expression of the astrocyte marker GFAP and the microglial marker Iba-1. The experimental results demonstrated (as depicted in Fig. 5A, B) that the expression levels of GFAP and Iba-1 in the hippocampus of mice in the model group were significantly elevated, indicating an active state of neuroinflammation. Following HLJDD intervention, the expression levels of Iba-1 and GFAP in the hippocampus of mice were significantly reduced, suggesting that HLJDD has a notable inhibitory effect on the activation of microglia and astrocytes. Furthermore, the mechanisms by which astrocytes and microglia contribute to neuroinflammation in Alzheimer’s disease are closely related to their subtypes. Therefore, we further analyzed the expression of different cell subtypes in the hippocampus of mice post-HLJDD intervention. Specifically, we examined the expression levels of type A1 astrocytes (GFAP^+^/C3^+^), type A2 astrocytes (GFAP^+^/S100A10^+^), type M1 microglia (Iba-1^+^/iNOS^+^), and type M2 microglia (Iba-1^+^/Arg-1^+^). The results indicated (as shown in Fig. 5A, B) that HLJDD could significantly inhibit the expression of GFAP^+^/C3^+^ and Iba-1^+^/iNOS^+^, while promoting the expression of GFAP^+^/S100A10 and Iba-1^+^/Arg-1^+^. This finding suggests that HLJDD may exert its anti-neuroinflammatory effects by modulating the subtype balance of astrocytes and microglia.

The permeability of BBB in patients with AD is elevated, allowing peripheral intrinsic and adaptive immune cells to infiltrate the brain. This infiltration of peripheral immune cells contributes to and mediates neuroinflammation, thereby significantly influencing the progression of AD. In this study, we observed marked infiltration of CD4^+^ and CD8^+^ T cells in the brains of mice in the model group. However, following intervention with HLJDD, the infiltration of CD4^+^ and CD8^+^ T cells in the mouse brains was significantly reduced (as illustrated in Fig. 5C). Additionally, we conducted a quantitative analysis of the expression levels of inflammatory factors in brain tissue, including pro-inflammatory factors IL-1β, IL-6, IFN-γ, and the anti-inflammatory factor IL-4. The results are presented in Fig. 5D. Compared to the model group, the HLJDD intervention led to a significant decrease in the expression levels of pro-inflammatory factors in the mouse brain, while the expression levels of anti-inflammatory factors increased, resulting in a notable reversal of the overall inflammatory state (*P* < 0.05). These findings suggest that HLJDD can effectively mitigate central nervous system inflammation in AD mice.

### Effects of HLJDD on peripheral inflammation in APP/PS1 mice

**Fig. 6:**
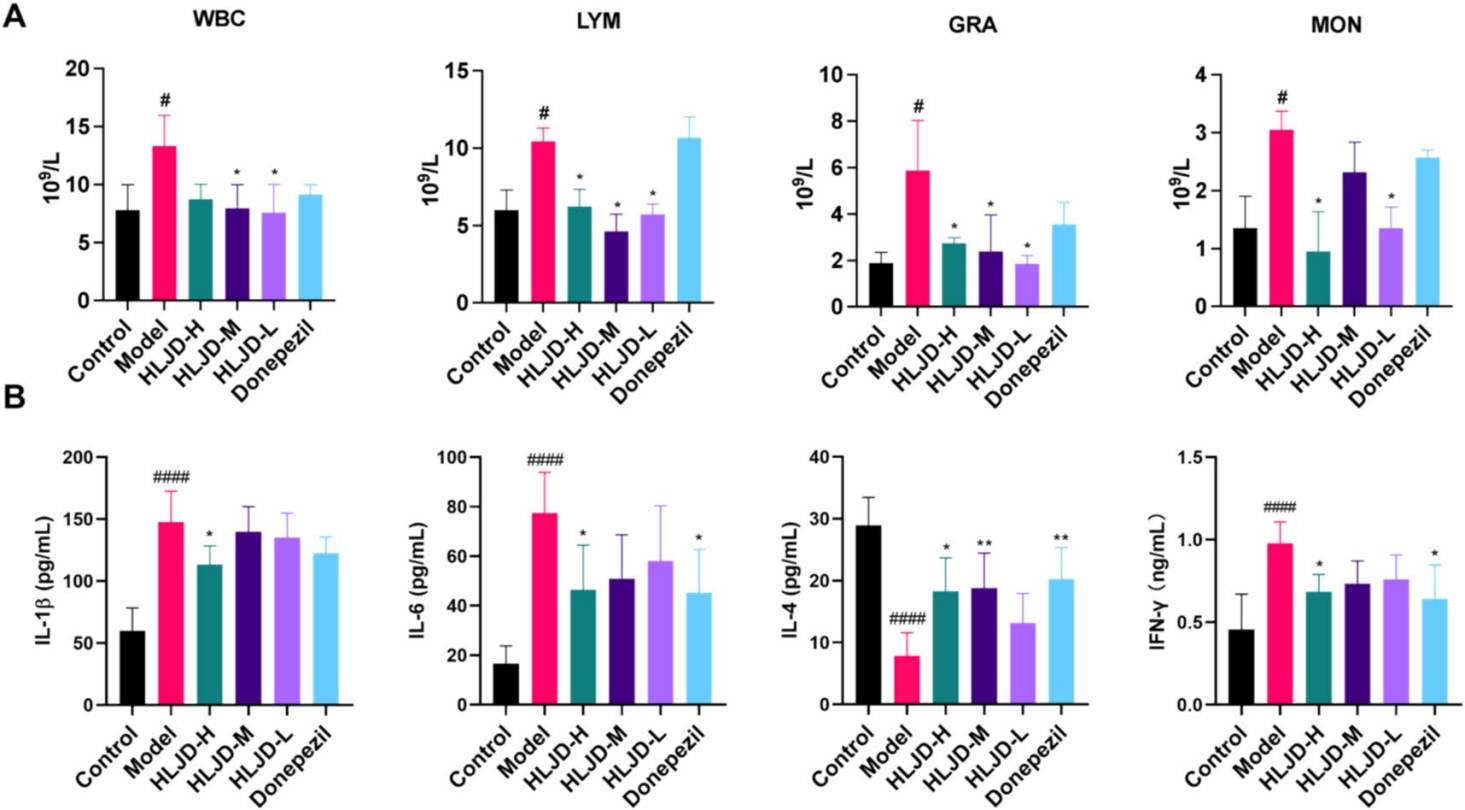
Effects of HLJDD on peripheral neuroinflammation in APP/PS1 mice. **A** Levels of IL-1β, IL-6, IL-4, and IFN-γ in peripheral serum. **B** Blood routine was used to detect the number of immune cells. Data are presented as mean ± SD (n = 6). ^#^*P* < 0.05, ^###^*P* < 0.001 and ^####^*P* < 0.0001 (vs control group), ^*^P < 0.05 and ^**^P < 0.01 (vs model group)

We further evaluated the improvement of peripheral inflammation following HLJDD intervention by analyzing the number of immune cells in the peripheral blood and the expression levels of inflammatory factors in the serum of mice across all groups. Our findings indicate that, compared to the blank group, the model group exhibited a significant increase in the number of immune cells in peripheral blood (Fig. 6A). Additionally, the expression levels of pro-inflammatory factors IL-1β, IL-6, and IFN-γ in serum were markedly elevated, while the expression level of the anti-inflammatory factor IL-4 was significantly reduced (Fig. 6B). This suggests an abnormal peripheral inflammatory response in AD mice. Following HLJDD intervention, a comparison with the model group revealed that the numbers of peripheral blood white blood cells, lymphocytes, monocytes, and granulocytes in the various HLJDD dose groups all decreased (Fig. 6A). Furthermore, the expression levels of peripheral blood inflammatory factors IL-1β, IL-6, IL-4, and IFN-γ in the HLJDD dose groups were all reversed compared to those in the model group (Fig. 6B). These results suggest that HLJDD exerts a significant inhibitory effect on the level of peripheral inflammation in AD mice.

### Effects of HLJDD on the intestinal barrier of APP/PS1 mice

**Fig. 7:**
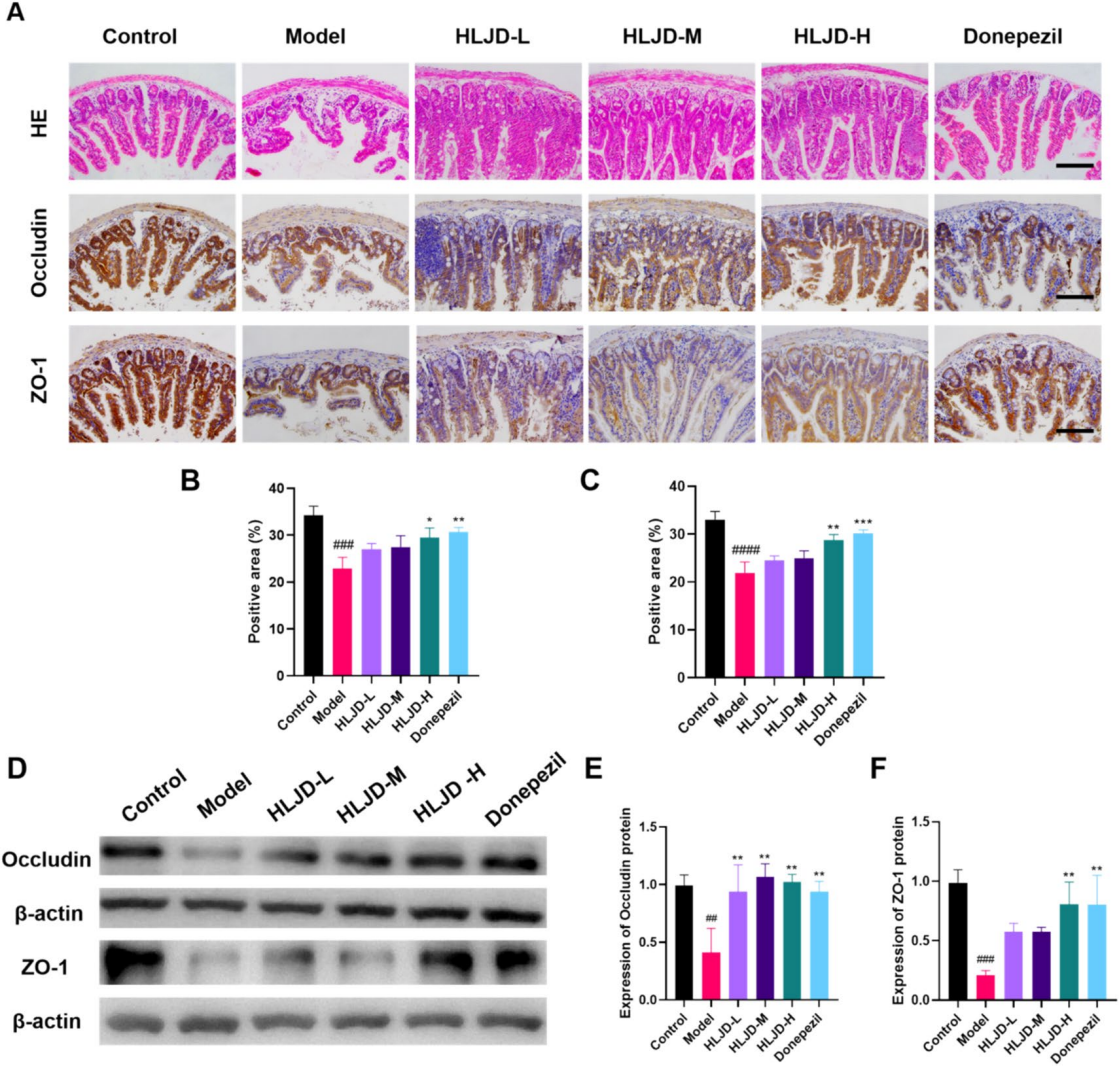
Effect of Huanglian-Jiedu decoction on intestinal barrier in APP/PS1 mice. **A** Representative image of HE staining in the mouse intestine, and representative images of immunohistochemistry of intestinal Occludin and ZO-1 (Scale bar = 50 μm). **B** Statistical analysis of the relative expression level of Occludin. **C** Statistical analysis of the relative expression level of ZO-1. **D** The expressions of ZO-1 and Occludin proteins in the intestine were detected by Western blotting. **E** Semi-quantitative analysis of the gray value of Occludin. **F** Semi-quantitative analysis of the gray value of ZO-1. Data are presented as mean ± SD (n = 3). ^##^*P* < 0.01 (vs control group), ^*^P < 0.05 and ^**^P < 0.01 (vs model group)

As shown in Fig. 7A, the structure of the small intestinal epithelium in the model group of mice was significantly disorganized and atrophied, however, the intestinal epithelial villi damage was improved by treatment with different concentrations of HLJDD. Immunohistochemical results showed that the expression of Occludin and ZO-1 was reduced in the model group compared with the control group, and improved after treatment. The results of western blot showed that the expression of Occludin and ZO-1 expression was downregulated in the model group compared to the control group, whereas the protein expression was upregulated after treatment (Fig. 7B–D, P < 0.05).

### Effects of HLJDD on gut microbiota in APP/PS1 mice

**Fig. 8:**
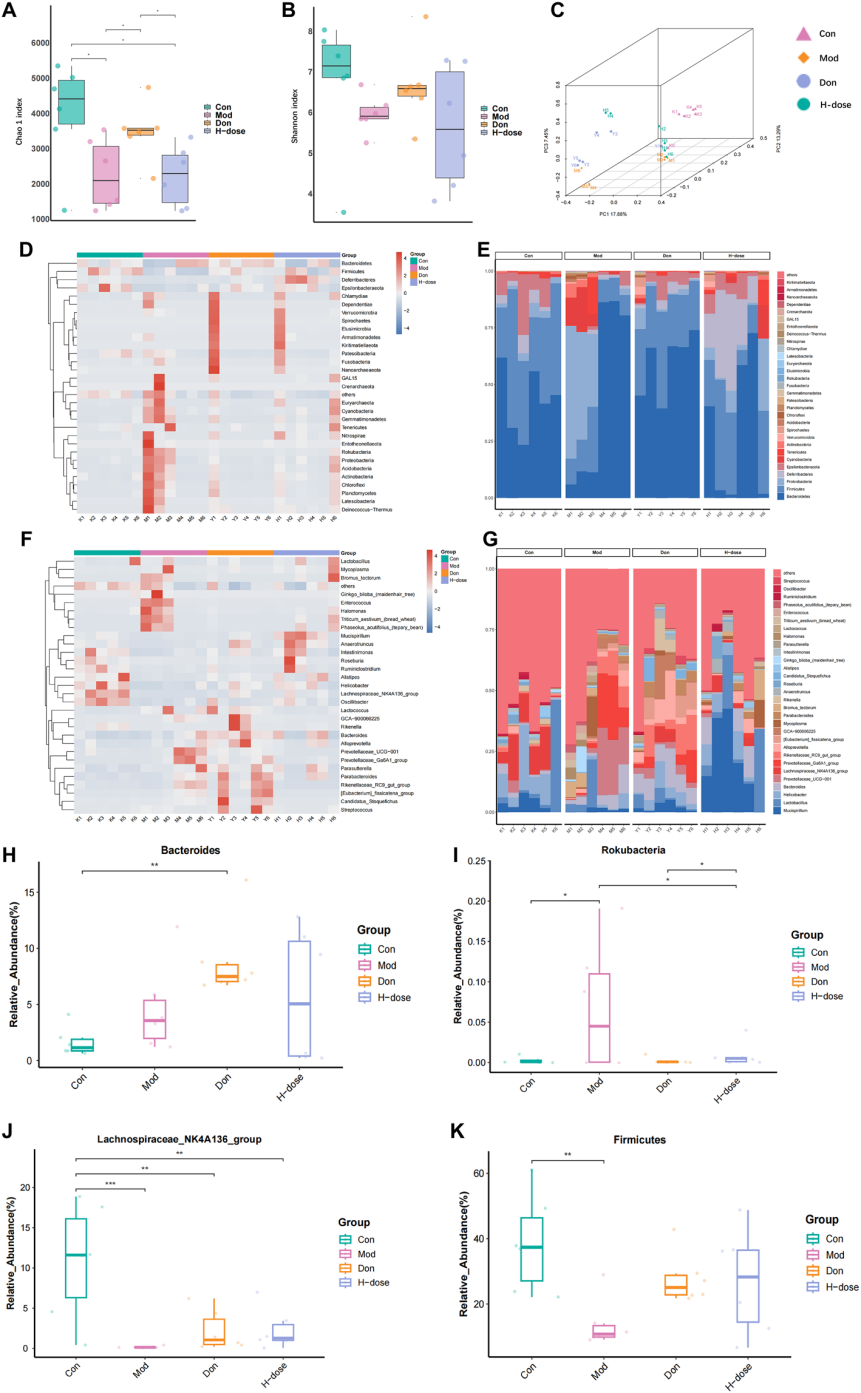
Effects of HLJDD on gut microbiota in APP/PS1 mice. **A** Chao 1 index.** B** Shannon index. **C** The principal co-ordinates analysis (PCoA) plot. **D** and **E** Heatmaps and histograms of gut microbiota composition at the phylum level (top 30 relative abundance). **F** and **G** Heatmaps and histograms gut microbiota composition at the genus level (top 30 relative abundance). **H–K** Relative abundance of Firmicutes, Rokubacteria, Bacteroides and Lachnospiraceae. Data are presented as mean ± SD (n = 6). ^*^P < 0.05, ^**^P < 0.01 and ^***^P < 0.001

The results of alpha and beta diversity analyses based on 16S rRNA gene sequencing showed that HLJDD had a significant effect on the composition of the gut microbiota. In terms of alpha diversity, changes in Chao1 index and Shannon index indicated that the model group varied from the treatment group in terms of the number of operational taxonomic units (OTUs) as well as the abundance and diversity of species (Fig. 8A, B). Beta diversity analyses further revealed that there were significant clustering differences in microbial taxonomic and functionality among the different groups through the PCOA three-dimensional maps (Fig. 8C). To estimate the distribution of mouse gut microbes in each group, we compared the relative abundance of the top 30 groups at the phylum and genus levels (Fig. 8D–G). At the phylum level, the abundance of the Firmicutes group in the model group was significantly reduced compared with the control group (*P* < 0.01), while the treatment group brought back its abundance, on the contrary, the abundance of the Rokubacteria group in the model group was significantly elevated (*P* < 0.05), and was treated and significantly decreased (Fig. 8H, I, P < 0.05). At the genus level, the abundance of Bacteroides was elevated but not statistically significant in the treatment group compared to the model group, however, the abundance of Lachnospiraceae decreased in the model group showed an upward trend after treatment (Fig. 8J, K). Taken together, these results suggest that HLJDD was able to modulate the structure of the gut microbiota and restore part of the disturbed microbial composition in the model group.

### Effects of HLJDD on the expression of inflammation-related proteins in APP/PS1 mice

**Fig. 9:**
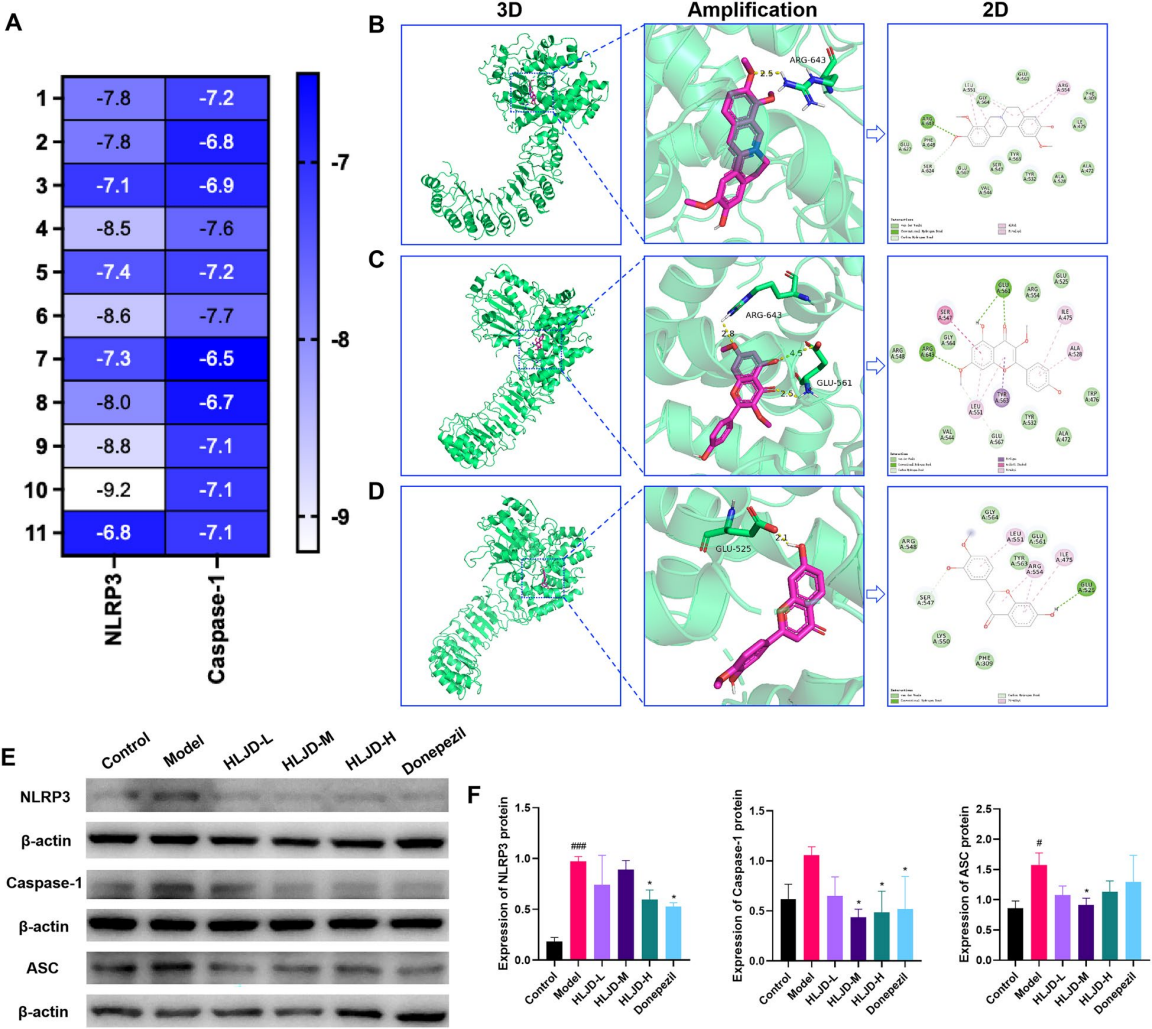
Effect of HLJDD on the expression of inflammation-related proteins in APP/PS1 mice. **A** Heat maps of the binding energies of each chemical component with NLRP3 and Caspase-1. **B**–**D** 3D and 2D interaction diagrams of the core compound and NLRP3 protein, including, **B** Jatrorrhizine, **C** Kumatakenin, **D** 3’,7-dihydroxy-4’-methoxyflavone. **E** The protein expression of NLRP3, Caspase-1 and ASC in the intestines was determined by Western blotting (n = 3).** F** grey-scale quantitative analysis of index. Data are presented as mean ± SD. ^#^*P* < 0.05 and ^###^*P* < 0.001 (vs control group), ^*^P < 0.05 (vs model group)

To further explore the interaction between HLJDD, NLRP3, and Caspase-1, we conducted a molecular docking analysis. Based on the degree value index, we screened the top-ranked active ingredients in HLJDD as core components for treating AD. The active ingredients include (12bs)−4,10,11-trimethoxy-7,8,12B,13-tetrahydro-5 h-6-azatetraphen-3-ol, Baicalein, Epiberberine, 3’,7-dihydroxy-4’-methoxyflavone, Thaliporphine, Wogonin, Kumatakenin, Jatrorrhizine, 6-ethyl-1,10-dimethoxy-5,6,6a,7-tetrahydro- 4H-dibenzo[de,g]quinolin-2,9-diol, phellodendrine, and Hamiltone A, which were molecularly docked with NLRP3 and Caspase-1, respectively. A binding energy threshold of −5 kcal/mol was considered indicative of good binding activity. The heat map of the docking results is shown in Fig. 9A. It was found that the binding energies of the compounds with NLRP3 were all lower than −5 kcal/mol, indicating that the main active components of HLJDD exhibit significant binding activity with NLRP3. Subsequently, the three complexes with the highest docking scores and binding energies were selected for further visualization (Fig. 9B–D). Next, we validated the expression of inflammation-related proteins in the intestines using Western blot (Fig. 9E). The expression of NLRP3, Caspase-1 and ASC proteins was significantly up-regulated in the model group compared to the control group, whereas HLJDD intervention significantly reversed these changes (Fig. 9F).

## Discussion

In this study, firstly, we used high-performance liquid chromatography (HPLC) fingerprinting and ultra-performance liquid chromatography-tandem quadrupole time-of-flight mass spectrometry (UPLC-Q-TOF/MS) to identify the exogenous and in vivo chemical constituents of HLJDD. Then we derived the relevant pathways of HLJDD for the treatment of AD including the inflammatory pathway through network pharmacology. Through the behavioral performance of mice in vivo, we found that HLJDD could effectively improve the symptoms of APP/PS1 mice, and we also found that HLJDD was also found to significantly reduce the proportion of M1 microglia and A1 astrocytes, while increasing the proportion of M2 microglia and A2 astrocytes, reducing central neuroinflammation and inhibiting Aβ deposition, in addition, it was found that HLJDD could reduce the expression of inflammatory factors in the brain and peripheral blood, and inhibit peripheral immune responses, the results of the gut microbiota showed that it was altered by the treatment of HLJDD, and the expression of NLRP3 inflammasome-related protein was also verified in the intestines. Therefore, it was hypothesized that HLJDD could improve the “central-peripheral” inflammatory microenvironment of APP/PS1 mice by inhibiting the activation of NLRP3 inflammasome mediated by the gut microbiota.

Recent studies have revealed that immune system disorders are not only secondary responses to AD, but also major participants in the occurrence and progression of the disease [21]. The body’s immune system includes the central immune system and the peripheral immune system. Microglia and astrocytes are the most important members of the central immune system, and play important regulatory roles in brain development, neuronal activity and neuroinflammation [22]. In the early stage of AD, activated microglia are able to remove and degrade Aβ by enhancing phagocytosis to prevent its aggregation [23]. After the further development of AD, due to the continuous stimulation of Aβ, microglia are repeatedly activated, and their phagocytosis of Aβ is gradually inhibited, and A variety of pro-inflammatory mediators are produced, which promote the production of Aβ, leading to the hyperphosphorylation of tau protein. At the same time, it also induces the differentiation of astrocytes into A1 phenotype and releases pro-inflammatory mediators. This results in an inflammatory cascade of Aβ deposition, repeated activation of glial cells, massive release of proinflammatory mediators, abnormal formation of Aβ, and hyperphosphorylation of tau, which eventually leads to dysregulation of the central immune system [24, 25]. Interestingly, we found that the related inflammatory factors in the peripheral blood of AD group mice were also increased. Studies have reported that a large number of peripheral inflammatory factors can cross the blood–brain barrier and further reach the central nervous system after entering the systemic circulation, and also cause central nervous system inflammation [15], which is exactly consistent with our results. In addition, HLJDD treatment can effectively eliminate Aβ, phosphorylated tau protein and other pathological products, inhibit the over-activation of microglia and astrocytes, inhibit the transformation of astrocytes and microglia into A1 and M1 types, and promote the transformation of A2 and M2 types, and block the infiltration of peripheral immune cells into the brain. Simultaneously improving the central and peripheral inflammatory microenvironment, thus effectively curbing the vicious cycle of"central and peripheral"immune interaction, providing a new intervention strategy for the treatment of AD.

The integrity of the intestinal barrier is a key factor in maintaining intestinal homeostasis, and its dysfunction is closely associated with the development of intestinal inflammation [26]. As the first line of defense of the intestinal internal environment, the intestinal epithelium and the mucus layer on its surface constitute a dynamic barrier separating the luminal contents from the submucosal neuroimmune system. In this barrier structure, tight junction proteins, such as ZO-1 protein and occludin, play a central role, which maintain the tight junctions of epithelial cells through the formation of intercellular junction complexes, thus ensuring the structural integrity and functional stability of the intestinal barrier [27–29]. Therefore, in this experiment, we examined the histopathology of the intestine and observed that the structure of the small intestinal epithelium in mice was significantly disorganized and atrophied, however, the intestinal epithelial villous damage was improved by treatment with different concentrations of HLJDD. In addition to this, the expression of the related proteins ZO-1 protein and occludin was examined, which showed that the expression of Occludin and ZO-1 was reduced in the model group compared to the blank group, and after treatment, the expression of Occludin and ZO-1 was elevated. Immediately following the treatment of HLJDD to determine whether dysregulation of gut ecology is associated with AD, we examined the fecal gut microbiota of APP/PS1 mice and their treated mice, and we found that Firmicute bacteria were significantly reduced in the model group compared to the blank group, and on the contrary, the treatment group elevated the abundance of this bacterial group. Rokubacteria were significantly elevated in the model group compared to the blank group, and the abundance of the bacteria was significantly reduced after treatment. In addition to this, the abundance of Bacteroides flora was elevated in the treatment group compared to the model group, but it was not significant, whereas the abundance of Lachnospiraceae flora was reduced in the model group compared to the blank group, which was elevated after treatment. It can be found that the alteration of gut microbiota composition is closely related to AD relationship.

Wasen et al. [30]. confirmed that Bacteroides play a significant role in regulating microglial function and influencing the onset of AD. This regulation is primarily achieved by enhancing the phagocytic activity of microglia, promoting the clearance of Aβ, and inhibiting the accumulation of amyloid plaques, thereby delaying the onset of AD. Wang et al. [31]. demonstrated that Gegen Qinlian tablets can inhibit the activation of microglia and astrocytes, mitigate neuroinflammatory responses, and significantly improve cognitive function in AD rat models. Furthermore, the mechanism of action is associated with the inhibition of the hippocampal NF-κB/MAPK signaling pathway and the restoration of abnormal levels of Firmicutes and Bacteroidetes. This study also confirmed that the levels of Firmicutes and Bacteroidetes within gut microbiota are closely related to the inflammatory response and cognitive function in AD. Additionally, Jun Fu et al. [32]. found in their research on the mechanisms through which Schisandra polysaccharides alleviate AD in rats that Firmicutes and Bacteroides are beneficial in delaying the progression of AD, with their expression decreasing in AD and increasing post-treatment. Moreover, a study examining the correlation between the microbiota-gut-brain axis and cognitive dysfunction in sporadic AD revealed that, compared to patients with cognitive impairment not caused by AD (CI-NAD), the abundance of Lachnospiraceae in the intestines of patients with cognitive impairment due to AD (CI-AD) was significantly reduced, which is believed to be linked to the cognitive dysfunction associated with AD, making it a potential biomarker [33]. Although there is currently no direct evidence suggesting that Rokubacteria are related to the inflammatory response in AD, considering the significant role of gut microbiota in the pathogenesis of AD, it can be theorized that any factor affecting the balance of gut microbiota may indirectly influence the inflammatory response in AD. In conclusion, Firmicutes, Bacteroidetes, Lachnospiraceae and Rokubacteria may serve as potential intestinal biomarkers for HLJDD in the context of improving AD.

In recent years, it has been found that dysregulation of gut microbiota can affect the function of the nervous system through multiple pathways, including secretion of metabolites, regulation of the neuroimmune system, and activation of pro-inflammatory signaling pathways. Among them, aberrant activation of the gut microbial-NLRP3 inflammatory vesicle pathway plays a key role in the neuroinflammatory response and the pathological process of AD [34, 35]. Studies have shown that NLRP3 promotes the release of pro-inflammatory cytokines such as IL-1β and IL-18 in the brain by inducing the activation of caspase-1, which further promotes the aggregation of intrinsic immune cells and initiates the downstream inflammatory cascade response, and ultimately accelerates the pathological progression of AD, which is a major mechanism of neuroinflammatory response [36–40]. therefore, we analyzed the expression of inflammasome-associated proteins in the present experiments to explore their role in neurodegenerative diseases, and we found that NLRP3, Caspase-1 and ASC protein expression was significantly upregulated in the AD group compared to the control group, while HLJDD intervention significantly reversed these changes. Overall, HLJDD can improve the central-peripheral inflammatory microenvironment and improve the cognitive function of APP/PS1 mice by inhibiting the activation of NLRP3 inflammasome mediated by gut microbiota.

## Conclusion

This study found that HLJDD could improve the neuroinflammatory microenvironment and enhance cognitive function by regulating the"central-peripheral"immune circuit in APP/PS1 mice, and at the same time, it could change the composition and balance of gut microbiota in AD mice. Further studies showed that the formula may mediate the neuroinflammatory response by activating the NLRP3 inflammasome pathway in the gut. These findings reveal the underlying mechanism of the link between gut and brain inflammation, and provide new intervention targets for the treatment of neurodegenerative diseases such as Alzheimer’s disease. However, the specific activation mechanism of inflammasomes in intestinal tissues is still unclear and needs to be further explored.

## Supplementary Information

Additional file 1.

Additional file 2.

Additional file 3.

Additional file 4.

Additional file 5.

## Data Availability

The data that support the findings of this study are available from the corresponding author, upon reasonable request.

## Abbreviations

AD: Alzheimer's disease
HLJDD: Huanglian Jiedu Decoction
TCM: Traditional Chinese medicine
UPLC-O-TOF/MS: Ultra-performance liquid chromatography-time-of-flight mass spectrometry
HPLC: High-performance liquid chromatography
NLRP3: NOD-like receptor protein 3
