# Supplementary material for: Huanglian Jiedu Decoction improves the"central-peripheral"inflammatory microenvironment and enhances the cognitive function of APP/PS1 mice by inhibiting the activation of NLRP3 inflammasome mediated by gut microbiota
Source: Chin Med. 2025 Aug 7;20:123. doi: 10.1186/s13020-025-01180-4 (PMC12330115; doi:10.1186/s13020-025-01180-4)
Supplement: Supplementary file 1 — Additional file 1. [file 13020_2025_1180_MOESM1_ESM.docx]

Tab. 1 The chemical components of Huanglian Jiedu Decoction

| NO | Formula | Molecular weight (Da) | Ionization model | Compounds |
| --- | --- | --- | --- | --- |
| 1 | C_20_H_23_NO_4_ | 341.1627 | [M+H]^+^ | (12bs)-4,10,11-trimethoxy-7,8,12b,13-tetrahydro-5h-6-azatetraphen-3-ol |
| 2 | C_16_H_20_O_10_ | 372.1056 | [M-H]^-^ | 1'-Galactoyl Ferulic acid |
| 3 | C_25_H_24_O_12_ | 516.1268 | [M-H]^-^ | Cynarin |
| 4 | C_10_H_22_O | 158.1671 | [M-H]^-^ | 1-Decanol |
| 5 | C_32_H_42_O_17_ | 698.2422 | [M-H]^-^ | 1-Hydroxypineolin Diglucoside |
| 6 | C_32_H_36_O_17_ | 692.1952 | [M-H]^-^ | 1-O-(6'-O-feruloyl)glucoside-3-O-Caffeoyl Quinic Acid |
| 7 | C_16_H_20_O_9_ | 356.1107 | [M-H]- | 1-O-Feruloyl-β-D-glucose |
| 8 | C_20_H_23_NO_4_ | 341.1627 | [M+H]^+^ | 10-(hydroxymethyl)-3,4-dimethoxy-7,8,12b,13-tetrahydro-5h-6-azatetraphen-11-ol |
| 9 | C_20_H_19_NO_4_ | 337.1314 | [M+H]^+^ | 13,13α-Didehydro-9,10-dimethoxy-2,3-(methylenedioxy)-berbine |
| 10 | C_17_H_14_O_7_ | 330.074 | [M+H]^+^ | Rehderianin I |
| 11 | C_20_H_17_NO_4_^+^ | 336.123 | [M]^+^ | berbine |
| 12 | C_30_H_48_O_5_ | 488.3502 | [M-H]^-^ | 2,3,23-Trihydroxyolean-12-en-28-oic acid |
| 13 | C_9_H_7_NO_2_ | 161.0477 | [M+H]^+^ | 2,4-Dihydroxyquinoline |
| 14 | C_20_H_23_NO_4_ | 342.1697 | [M+H]^+^ | phellodendrine |
| 15 | C_8_H_8_O_4_ | 168.0423 | [M+H]^+^ | 2,6-Dimethoxy-1,4-benzoquinone |
| 16 | C_10_H_22_O | 158.1671 | [M-H]^-^ | 2-Decanol |
| 17 | C_10_H_9_NO_3_ | 191.0582 | [M+H]^+^ | 2-Oxo-3,4-dihydro-1H-quinoline-3-carboxylic acid |
| 18 | C_22_H_24_O_12_ | 480.1268 | [M+H]^+^ | 3',5',5,7-Tetrahydroxy-4'-methoxyflavanone-3'-O-glucoside |
| 19 | C_16_H_12_O_5_ | 284.0685 | [M+H]^+^ | 3',7-dihydroxy-4'-methoxyflavone |
| 20 | C_26_H_30_O_13_ | 550.1686 | [M-H]^-^ | 3'-O-Feruloyl Swertiamarin |
| 21 | C_21_H_22_O_11_ | 450.1162 | [M-H]^-^ | 3,4,2',4',6'-Pentahydroxychalcone-4'-O-glucoside |
| 22 | C_25_H_32_O_13_ | 540.1843 | [M-H]^-^ | 3,4-Dihydro-6-O-feruloylcatalpol |
| 23 | C_7_H_6_O_4_ | 154.0266 | [M-H]^-^ | Protocatechuic acid |
| 24 | C_16_H_18_O_8_ | 338.1002 | [M-H]^-^ | 3-(Hydroxycinnamoyl)-quinic acid |
| 25 | C_17_H_20_O_9_ | 368.1107 | [M+H]^+^ | 3-O-Feruloylquinic acid |
| 26 | C_26_H_26_O_12_ | 530.1424 | [M+H]^+^ | 3-O-caffeioyl feruloyl quinic acid |
| 27 | C_9_H_10_O_2_ | 150.0681 | [M+H]^+^ | 4'-Methoxyacetophenone |
| 28 | C_9_H_7_NO_2_ | 161.0477 | [M+H]^+^ | 4,6-Dihydroxyquinoline |
| 29 | C_8_H_8_O_2_ | 136.0524 | [M-H]^-^ | 4-Hydroxyacetophenone |
| 30 | C_22_H_28_O_14_ | 516.1479 | [M-H]^-^ | 4-O-(3'-O-alpha-D-Glucopyranosyl)caffeoylquinic acid |
| 31 | C_22_H_28_O_14_ | 516.1479 | [M-H]^-^ | 4-O-(4'-O-alpha-D-Glucopyranosyl)caffeoylquinic acid |
| 32 | C_16_H_20_O_9_ | 356.1107 | [M-H]^-^ | 4-O-β-D-glucopyranosylferulic acid |
| 33 | C_16_H_16_O_8_ | 336.0845 | [M-H]^-^ | 4-caffeoylshikimic acid |
| 34 | C_16_H_14_O_5_ | 286.0841 | [M-H]^-^ | 5,2'-Dihydroxy-7-methoxyflavanone |
| 35 | C_17_H_14_O_6_ | 314.079 | [M-H]^-^ | Kumatakenin |
| 36 | C_19_H_20_O_6_ | 344.126 | [M+H]^+^ | 5,6,7,4'-Tetramethoxyflavanone |
| 37 | C_20_H_22_O_7_ | 374.1366 | [M+H]^+^ | 5,7,8,3',4'-Pentamethoxyflavanone |
| 38 | C_10_H_9_NO_3_ | 191.0582 | [M-H]^-^ | 5-Hydroxyindole-3-acetic acid |
| 39 | C_6_H_6_O_3_ | 126.0317 | [M+H]^+^ | 5-Hydroxymethylfurfural |
| 40 | C_6_H_6_O_3_ | 126.0317 | [M+H]^+^ | 5-Methoxyfurfural |
| 41 | C_34_H_44_O_19_ | 756.2477 | [M+H]^+^ | 6''-O-Trans-Sinapoylgenipin gentiobioside |
| 42 | C_32_H_40_O_17_ | 696.2265 | [M+NH_4_]^+^ | 6''-O-p-Coumaroylgenipin gentiobioside |
| 43 | C_28_H_34_O_14_ | 594.1949 | [M+H]^+^ | 6'-O-Sinapoylgeniposide |
| 44 | C_27_H_32_O_14_ | 580.1792 | [M+H]^+^ | 6'-O-[(E)-sinapoyl]gardoside |
| 45 | C_22_H_22_O_11_ | 462.1162 | [M+H]^+^ | 6-C-MethylKaempferol-3-glucoside |
| 46 | C_15_H_22_O_9_ | 346.1264 | [M-H]^-^ | 6-DeoxyCatalpol |
| 47 | C_18_H_18_O_6_ | 330.1103 | [M-H]^-^ | Hamiltone A |
| 48 | C_21_H_20_O_12_ | 464.0955 | [M+H]^+^ | 6-Hydroxykaempferol-7-O-glucoside |
| 49 | C_20_H_23_NO_4_ | 341.1627 | [M+H]^+^ | 6-ethyl-1,10-dimethoxy-5,6,6a,7-tetrahydro-4H-dibenzo[de,g]quinoline-2,9-diol |
| 50 | C_9_H_7_NO | 145.0528 | [M+H]^+^ | 8-hydroxyquinoline |
| 51 | C_28_H_32_O_14_ | 592.1792 | [M+H]^+^ | Linarin |
| 52 | C_27_H_30_O_14_ | 578.1636 | [M+H]^+^ | Isorhoifolin |
| 53 | C_15_H_10_O_5_ | 270.0528 | [M+H]^+^ | Baicalein |
| 54 | C_21_H_18_O_11_ | 446.0849 | [M+H]^+^ | Baicalin |
| 55 | C_9_H_8_O_4_ | 180.0423 | [M-H]^-^ | Caffeic acid |
| 56 | C_9_H_8_O_3_ | 164.0473 | [M+H]^+^ | Caffeic aldehyde |
| 57 | C_16_H_12_O_5_ | 284.0685 | [M+H]^+^ | Calycosin |
| 58 | C_22_H_22_O_10_ | 446.1213 | [M+H]^+^ | Calycosin-7-O-glucoside |
| 59 | C_30_H_48_O_5_ | 488.3502 | [M-H]^-^ | Cannabifolin C |
| 60 | C_16_H_18_O_9_ | 354.0951 | [M-H]^-^ | Chlorogenic acid |
| 61 | C_17_H_20_O_9_ | 368.1107 | [M-H]^-^ | Chlorogenic acid methyl ester |
| 62 | C_15_H_10_O_4_ | 254.0579 | [M+H]^+^ | Chrysin |
| 63 | C_27_H_30_O_14_ | 578.1636 | [M+H]^+^ | Chrysin-5,7-Di-O-glucoside |
| 64 | C_21_H_20_O_9_ | 416.1107 | [M+H]^+^ | Chrysin-5-O-glucoside (Toringin) |
| 65 | C_26_H_28_O_13_ | 548.153 | [M+H]^+^ | Chrysin-6-C-arabinoside-8-C-glucoside |
| 66 | C_24_H_22_O_12_ | 502.1111 | [M+H]^+^ | Chrysin-7-O-(6'-malonyl)glucoside |
| 67 | C_21_H_18_O_10_ | 430.09 | [M+H]^+^ | Chrysin-7-O-Glucuronide |
| 68 | C_15_H_16_O_9_ | 340.0794 | [M-H]^-^ | Cichoriin |
| 69 | C_30_H_48_O_5_ | 488.3502 | [M-H]^-^ | Cordianol B |
| 70 | C_19_H_16_NO_4_ | 322.1074 | [M+H]^+^ | berberrubine |
| 71 | C_19_H_18_NO_4_ | 324.123 | [M+H]^+^ | Demethylene berberine |
| 72 | C_19_H_13_NO_4_ | 320.0917 | [M+H]^+^ | coptisine |
| 73 | C_20_H_24_O_4_ | 328.1675 | [M-H]^-^ | Crocetin |
| 74 | C_44_H_64_O_24_ | 976.3788 | [M-H]^-^ | Crocin |
| 75 | C_44_H_64_O_24_ | 976.3788 | [M-H]^-^ | Crocin I |
| 76 | C_6_H_10_O_2_ | 114.0681 | [M+H]^+^ | Delta-Hexalactone |
| 77 | C_21_H_24_O_10_ | 436.1369 | [M-H]^-^ | Dihydrocharcone-4'-O-glucoside |
| 78 | C_16_H_14_O_6_ | 302.079 | [M+H]^+^ | Dihydrokaempferide |
| 79 | C_21_H_22_O_11_ | 450.1162 | [M+H]^+^ | Dihydrokaempferol-3-O-glucoside |
| 80 | C_12_H_16_O_4_ | 224.1049 | [M-H_2_O+H]^+^ | E-6,7-Dihydroxydihydroligustilide |
| 81 | C_20_H_18_NO_4_ | 336.123 | [M]^+^ | Epiberberine |
| 82 | C_15_H_14_O_6_ | 290.079 | [M+H]^+^ | Epicatechin |
| 83 | C_22_H_18_O_10_ | 442.09 | [M-H]^-^ | Epicatechin gallate |
| 84 | C_30_H_26_O_13_ | 594.1373 | [M+H]^+^ | Epicatechin-(4β→8)-epigallocatechin |
| 85 | C_20_H_22_O_6_ | 358.1416 | [M-H]^-^ | Epipinoresinol |
| 86 | C_17_H_24_O_11_ | 404.1319 | [M+NH_4_^]+^ | Gardenoside |
| 87 | C_22_H_32_O_15_ | 536.1741 | [M-H]^-^ | Gardenoside-Arabinoside |
| 88 | C_23_H_34_O_16_ | 566.1847 | [M-H]^-^ | Gardenoside-glucoside |
| 89 | C_16_H_22_O_10_ | 374.1213 | [M-H]^-^ | Gardoside |
| 90 | C_11_H_14_O_5_ | 226.0841 | [M+H]^+^ | Genipin |
| 91 | C_22_H_32_O_14_ | 520.1792 | [M+H]^+^ | Genipin-1-O-(2''-O-apiosyl)glucoside |
| 92 | C_23_H_34_O_15_ | 550.1898 | [M-H]^-^ | Genipin-1-O-gentiobioside |
| 93 | C_17_H_24_O_10_ | 388.1369 | [M+H]^+^ | Geniposide |
| 94 | C_16_H_22_O_10_ | 374.1213 | [M-H]^-^ | Geniposidic acid |
| 95 | C_21_H_22_O_10_ | 434.1213 | [M-H]^-^ | Hydroxy isoliquiritigenin glucoside |
| 96 | C_10_H_14_O_2_ | 166.0994 | [M+H]^+^ | Hydroxycarvine |
| 97 | C_11_H_18_O_3_ | 198.1256 | [M-H]^-^ | Hydroxydihydrobovolide |
| 98 | C_26_H_30_O_9_ | 486.189 | [M+H]^+^ | Isobavachalcone glucoside |
| 99 | C_25_H_24_O_12_ | 516.1268 | [M-H]^-^ | Isochlorogenic acid A |
| 100 | C_25_H_24_O_12_ | 516.1268 | [M-H]^-^ | Isochlorogenic acid C |
| 101 | C_10_H_10_O_4_ | 194.0579 | [M-H]^-^ | Isoferulic Acid |
| 102 | C_12_H_22_O_11_ | 342.1162 | [M-H]^-^ | Isomaltulose |
| 103 | C_12_H_18_O_3_ | 210.1256 | [M-H]^-^ | Jasmonic acid |
| 104 | C_20_H_20_NO_4_^+^ | 338.1387 | [M]^+^ | Jatrorrhizine |
| 105 | C_15_H_10_O_6_ | 286.0477 | [M-H]^-^ | Kaempferol |
| 106 | C_27_H_30_O_15_ | 594.1585 | [M+H]^+^ | Luteolin-7-O-rutinoside |
| 107 | C_22_H_28_O_8_ | 420.1784 | [M-H]^-^ | Lyoniresinol |
| 108 | C_21_H_22_O_10_ | 434.1213 | [M-H]^-^ | Prunin |
| 109 | C_16_H_18_O_9_ | 354.0951 | [M-H]^-^ | Neochlorogenic acid |
| 110 | C_23_H_24_O_13_ | 508.1217 | [M+H]^+^ | Okanin-4'-(6''-O-acetyl)glucoside |
| 111 | C_21_H_20_O_10_ | 432.1056 | [M+H]^+^ | Oroxin A |
| 112 | C26H30O_11_ | 518.1788 | [M+H]^+^ | Phellamurin |
| 113 | C_26_H_30_O_12_ | 534.1737 | [M-H]^-^ | Phellatin |
| 114 | C_26_H_32_O_12_ | 536.1894 | [M-H]^-^ | Phellavin |
| 115 | C_26_H_30_O_11_ | 518.1788 | [M+H]^+^ | Phellodendroside |
| 116 | C_15_H_12_O_4_ | 256.0736 | [M-H]^-^ | Dihydrochrysin |
| 117 | C_20_H_22_O_6_ | 358.1416 | [M-H]^-^ | Pinoresinol |
| 118 | C_16_H_14_O_4_ | 270.0892 | [M+H]^+^ | Pinostrobin Chalcone |
| 119 | C_8_H8O_4_ | 168.0423 | [M-H]^-^ | Protocatechuic Acid Methyl Ester |
| 120 | C_19_H_26_O_13_ | 462.1373 | [M-H]^-^ | Protocatechuic acid 1-O-Rutinoside |
| 121 | C_27_H_30_O_16_ | 610.1534 | [M-H]^-^ | Rutin |
| 122 | C_12_H_12_O_3_ | 204.0786 | [M+H]^+^ | Senkyunolide B |
| 123 | C_16_H_22_O_4_ | 278.1518 | [M+H]^+^ | Senkyunolide M |
| 124 | C_28_H_32_O_16_ | 624.169 | [M+H]^+^ | Sexangularetin-3-O-glucoside-7-O-rhamnoside |
| 125 | C_18_H_16_O_8_ | 360.0845 | [M-H]^-^ | Sudachitin |
| 126 | C_20_H_20_O_7_ | 372.1209 | [M+H]^+^ | Tangeretin |
| 127 | C_18_H_16_O_7_ | 344.0896 | [M+H]^+^ | Tenaxin I |
| 128 | C_20_H_23_NO_4_ | 341.1627 | [M+H]^+^ | Thaliporphine |
| 129 | C_27_H_34_O_12_ | 550.205 | [M-H]^-^ | Tracheloside |
| 130 | C_21_H_18_O_13_ | 478.0747 | [M-H]^-^ | Tricetin 3'-glucuronide |
| 131 | C_16_H_12_O_5_ | 284.0685 | [M+H]^+^ | Wogonin |
| 132 | C_22_H_20_O_11_ | 460.1006 | [M+H]^+^ | Wogonoside |
| 133 | C_16_H_17_NO_9_ | 367.0903 | [M-H]^-^ | Xanthurenic Acid 8-O-Glucoside |
| 134 | C_21_H_22_NO_4_^+^ | 352.1543 | [M]^+^ | Yanhusuine |
| 135 | C_36_H_36_O_19_ | 772.1851 | [M-H]^-^ | kaempferol-3-caffeoyldiglucoside |
| 136 | C_9_H_7_NO | 145.0528 | [M+H]^+^ | Α-hydroxyquinoline |
| 137 | C_21_H_26_O_4_ | 342.1831 | [M-H]^-^ | β-Crocetin |
