# Supplementary material for: Huanglian Jiedu Decoction improves the"central-peripheral"inflammatory microenvironment and enhances the cognitive function of APP/PS1 mice by inhibiting the activation of NLRP3 inflammasome mediated by gut microbiota
Source: Chin Med. 2025 Aug 7;20:123. doi: 10.1186/s13020-025-01180-4 (PMC12330115; doi:10.1186/s13020-025-01180-4)
Supplement: Supplementary file 3 — Additional file 3. [file 13020_2025_1180_MOESM3_ESM.docx]

Tab. S3 Initial identification of compounds into the blood

| NO | Formula | Molecular weight (Da) | Ionization model | Compounds |
| --- | --- | --- | --- | --- |
| 1 | C_20_H_23_NO_4_ | 341.1627 | [M+H]^+^ | (12bs)-4,10,11-trimethoxy-7,8,12b,13-tetrahydro-5h-6-azatetraphen-3-ol |
| 2 | C_25_H_24_O_12_ | 516.1268 | [M-H]^-^ | Cynarin |
| 3 | C_16_H_20_O_9_ | 356.1107 | [M-H]- | 1-O-Feruloyl-β-D-glucose |
| 4 | C_20_H_23_NO_4_ | 341.1627 | [M+H]^+^ | 10-(hydroxymethyl)-3,4-dimethoxy-7,8,12b,13-tetrahydro-5h-6-azatetraphen-11-ol |
| 5 | C_20_H_19_NO_4_ | 337.1314 | [M+H]^+^ | 13,13α-Didehydro-9,10-dimethoxy-2,3-(methylenedioxy)-berbine |
| 6 | C_20_H_17_NO_4_^+^ | 336.123 | [M]^+^ | berbine |
| 7 | C_20_H_23_NO_4_ | 342.1697 | [M+H]^+^ | phellodendrine |
| 8 | C_16_H_12_O_5_ | 284.0685 | [M+H]^+^ | 3',7-dihydroxy-4'-methoxyflavone |
| 9 | C_26_H_30_O_13_ | 550.1686 | [M-H]^-^ | 3'-O-Feruloyl Swertiamarin |
| 10 | C_25_H_32_O_13_ | 540.1843 | [M-H]^-^ | 3,4-Dihydro-6-O-feruloylcatalpol |
| 11 | C_17_H_20_O_9_ | 368.1107 | [M+H]^+^ | 3-O-Feruloylquinic acid |
| 12 | C_17_H_14_O_6_ | 314.079 | [M-H]^-^ | Kumatakenin |
| 13 | C_10_H_9_NO_3_ | 191.0582 | [M-H]^-^ | 5-Hydroxyindole-3-acetic acid |
| 14 | C_32_H_40_O_17_ | 696.2265 | [M+NH_4_]^+^ | 6''-O-p-Coumaroylgenipin gentiobioside |
| 15 | C_22_H_22_O_11_ | 462.1162 | [M+H]^+^ | 6-C-MethylKaempferol-3-glucoside |
| 16 | C_15_H_22_O_9_ | 346.1264 | [M-H]^-^ | 6-DeoxyCatalpol |
| 17 | C_18_H_18_O_6_ | 330.1103 | [M-H]^-^ | Hamiltone A |
| 18 | C_20_H_23_NO_4_ | 341.1627 | [M+H]^+^ | 6-ethyl-1,10-dimethoxy-5,6,6a,7-tetrahydro-4H-dibenzo[de,g]quinoline-2,9-diol |
| 19 | C_15_H_10_O_5_ | 270.0528 | [M+H]^+^ | Baicalein |
| 20 | C_21_H_18_O_11_ | 446.0849 | [M+H]^+^ | Baicalin |
| 21 | C_9_H_8_O_4_ | 180.0423 | [M-H]^-^ | Caffeic acid |
| 22 | C_9_H_8_O_3_ | 164.0473 | [M+H]^+^ | Caffeic aldehyde |
| 23 | C_16_H_18_O_9_ | 354.0951 | [M-H]^-^ | Chlorogenic acid |
| 24 | C_17_H_20_O_9_ | 368.1107 | [M-H]^-^ | Chlorogenic acid methyl ester |
| 25 | C_26_H_28_O_13_ | 548.153 | [M+H]^+^ | Chrysin-6-C-arabinoside-8-C-glucoside |
| 26 | C_21_H_18_O_10_ | 430.09 | [M+H]^+^ | Chrysin-7-O-Glucuronide |
| 27 | C_20_H_24_O_4_ | 328.1675 | [M-H]^-^ | Crocetin |
| 28 | C_44_H_64_O_24_ | 976.3788 | [M-H]^-^ | Crocin |
| 29 | C_44_H_64_O_24_ | 976.3788 | [M-H]^-^ | Crocin I |
| 30 | C_12_H_16_O_4_ | 224.1049 | [M-H_2_O+H]^+^ | E-6,7-Dihydroxydihydroligustilide |
| 31 | C_20_H_18_NO_4_ | 336.123 | [M]^+^ | Epiberberine |
| 32 | C_15_H_14_O_6_ | 290.079 | [M+H]^+^ | Epicatechin |
| 33 | C_22_H_18_O_10_ | 442.09 | [M-H]^-^ | Epicatechin gallate |
| 34 | C_16_H_22_O_10_ | 374.1213 | [M-H]^-^ | Gardoside |
| 35 | C_23_H_34_O_15_ | 550.1898 | [M-H]^-^ | Genipin-1-O-gentiobioside |
| 36 | C_17_H_24_O_10_ | 388.1369 | [M+H]^+^ | Geniposide |
| 37 | C_16_H_22_O_10_ | 374.1213 | [M-H]^-^ | Geniposidic acid |
| 38 | C_25_H_24_O_12_ | 516.1268 | [M-H]^-^ | Isochlorogenic acid A |
| 39 | C_25_H_24_O_12_ | 516.1268 | [M-H]^-^ | Isochlorogenic acid C |
| 40 | C_20_H_20_NO_4_^+^ | 338.1387 | [M]^+^ | Jatrorrhizine |
| 41 | C_16_H_18_O_9_ | 354.0951 | [M-H]^-^ | Neochlorogenic acid |
| 42 | C_23_H_24_O_13_ | 508.1217 | [M+H]^+^ | Okanin-4'-(6''-O-acetyl)glucoside |
| 43 | C_21_H_20_O_10_ | 432.1056 | [M+H]^+^ | Oroxin A |
| 44 | C_27_H_30_O_16_ | 610.1534 | [M-H]^-^ | Rutin |
| 45 | C_20_H_23_NO_4_ | 341.1627 | [M+H]^+^ | Thaliporphine |
| 46 | C_16_H_12_O_5_ | 284.0685 | [M+H]^+^ | Wogonin |
| 47 | C_22_H_20_O_11_ | 460.1006 | [M+H]^+^ | Wogonoside |
| 48 | C_21_H_26_O_4_ | 342.1831 | [M-H]^-^ | β-Crocetin |
